# O-GlcNAcylation of STAT5 controls tyrosine phosphorylation and oncogenic transcription in STAT5-dependent malignancies

**DOI:** 10.1038/leu.2017.4

**Published:** 2017-02-10

**Authors:** P Freund, M A Kerenyi, M Hager, T Wagner, B Wingelhofer, H T T Pham, M Elabd, X Han, P Valent, F Gouilleux, V Sexl, O H Krämer, B Groner, R Moriggl

**Affiliations:** 1 Ludwig Boltzmann Institute for Cancer Research, Vienna, Austria; 2 Department of Biomedical Science, Institute of Animal Breeding and Genetics, University of Veterinary Medicine, Vienna, Austria; 3 Department of Pharmacology, Boehringer Ingelheim RCV GmbH & Co KG, Vienna, Austria; 4 Department of Biochemistry, Center for Molecular Biomedicine, Friedrich Schiller University Jena, Jena, Germany; 5 Division of Gastroenterology, Hepatology and Nutrition, Cincinnati Children's Hospital Medical Center, Cincinnati, OH, USA; 6 Key Laboratory of Human Disease Comparative Medicine, Ministry of Health; Institute of Laboratory Animal Science, Chinese Academy of Medical Sciences (CAMS) and Peking Union Medical College (PUMC), Beijing, China; 7 Department of Internal Medicine I, Division Hematology and Hemostaseology and Ludwig Boltzmann Cluster Oncology, Medical University of Vienna, Vienna, Austria; 8 CNRS UMR 7292, Université François Rabelais, Tours, France; 9 Department of Biomedical Science, Institute of Pharmacology and Toxicology, University of Veterinary Medicine, Vienna, Austria; 10 Department of Toxicology, University Medical Center, Mainz, Germany; 11 Georg Speyer Haus, Institute for Tumor Biology and Experimental Therapy, Frankfurt am Main, Germany; 12 Medical University of Vienna, Vienna, Austria

## Abstract

The signal transducer and activator of transcription 5 (STAT5) regulates differentiation, survival, proliferation and transformation of hematopoietic cells. Upon cytokine stimulation, STAT5 tyrosine phosphorylation (pYSTAT5) is transient, while in diverse neoplastic cells persistent overexpression and enhanced pYSTAT5 are frequently found. Post-translational modifications might contribute to enhanced STAT5 activation in the context of transformation, but the strength and duration of pYSTAT5 are incompletely understood. We found that O-GlcNAcylation and tyrosine phosphorylation act together to trigger pYSTAT5 levels and oncogenic transcription in neoplastic cells. The expression of a mutated hyperactive gain-of-function (GOF) STAT5 without O-GlcNAcylation resulted in decreased tyrosine phosphorylation, oligomerization and transactivation potential and complete loss of oncogenic transformation capacity. The lack of O-GlcNAcylation diminished phospho-ERK and phospho-AKT levels. Our data show that O-GlcNAcylation of STAT5 is an important process that contributes to oncogenic transcription through enhanced STAT5 tyrosine phosphorylation and oligomerization driving myeloid transformation. O-GlcNAcylation of STAT5 could be required for nutrient sensing and metabolism of cancer cells.

## Introduction

The signal transducer and activator of transcription 5A (STAT5A) and STAT5B transcription factors have a conserved functional domain structure organized in N-terminal oligomerization, Coiled-coil, DNA-binding, Linker, SH2 and C-terminal transactivation domain.^1, 2^ Phosphorylation of a critical tyrosine residue at position 694/699 of STAT5A/B triggers the activation process.^3^ STAT5 regulated signaling controls important functions of innate and acquired immunity, hematopoiesis, and also growth and survival of many cell types in different organs responding to extracellular cytokines.^4, 5, 6^ STAT5 signaling also has a key role in human cancers where frequent mutations and hyperactivation promote enhanced activation or reduced inactivation.^5, 6^

Cytokine binding to the cell surface leads to phosphorylation of the receptor complex by receptor associated Janus kinases (JAKs) at tyrosine residues.^7^ The JAKs also trigger the activation and tyrosine phosphorylation of STAT5, causing parallel dimerization, nuclear translocation and tetramerization of STAT5.^7, 8^ Additionally, phosphorylation of serine 725 of STAT5A promotes transcriptional elongation, whereas phosphorylation of serine 779 by the FAK-PAK/RHO-RAC signaling cascade is required for nuclear translocation.^9, 10^ The deregulation of the extent and duration of STAT5 activation results in persistent tyrosine and high serine phosphorylation. Both are linked to transformation due to deregulated kinase/phosphatase signaling or STAT5 gain-of-function (GOF) mutations.^9, 11, 12, 13^

Additional post-translational modifications, such as ubiquitination, sumoylation, acetylation and glycosylation have been detected in STAT5.^14^ Interestingly, >4000 proteins are modified by O-linked β-D-N-acetylglucosamine (O-GlcNAc).^15^ The dynamic addition and removal of O-GlcNAc has been described for multiple cytoplasmic and nuclear proteins and it affects the function of various signaling molecules and transcription factors.^16^ The cycling of O-GlcNAc is dependent upon UDP-GlcNAc, synthesized in the hexosamine biosynthesis pathway,^17^ which serves as substrate for the O-GlcNAc transferase (OGT). OGT transfers O-GlcNAc moieties from UDP-GlcNAc to serine and threonine residues of proteins and O-GlcNAcase (OGA) catalyzes the opposite reaction to remove O-GlcNAc.^18^ Both enzymes, OGT and OGA as well as multiple O-GlcNAcylated proteins are enriched at sites of active transcription in human cells. O-GlcNAcylation regulates the transcriptional activity of p53, c-Myc, FoxO1 and CREB, as well as components of the basal transcription machinery, for example, the C-terminal domain of RNA polymerase II.^16^ Cancer cells exhibit elevated levels of O-GlcNAc, possibly to adjust to stringent metabolic demands and O-GlcNAcylation is a key metabolic regulator of glucose metabolism.^17, 18^

Here, we addressed the biological function of O-GlcNAcylation of STAT5. We characterized glycosylation deficient and/or hyperactive STAT5 variants and compared their oncogenic potential. The inability of STAT5 to be O-GlcNAcylated resulted in impaired tyrosine phosphorylation and a decrease of its transactivation potential. Moreover, hyperactive GOF STAT5A proteins increased phosphatidylinositol 3-kinase (PI3K)-AKT and ERK kinase activity in a glycosylation-dependent manner.

## Materials and methods

### Structural analysis

The human STAT5 N-terminal (accession number P42229) sequence was obtained from The Universal Protein Resource (UniProt).^19^ The positions of the eight alpha-helices in STAT5 were modeled based on the STAT4 N-terminal structure using Swissmodel^20, 21, 22, 23^ (http://swissmodel.expasy.org/). The crystal structure was obtained from the RSCB Protein Data Bank (www.rcsb.org)^24^ PDB ID 1BGF^25^ and colored using ADOBE PHOTOSHOP CS5 EXTENDED Version 12.0.4 x32 (Adobe Systems, San José, CA, USA). For the prediction of phosphorylation sites, PhosphoSitePlus (www.phosphosite.org) was used.^26^

### Gene transfer vectors, bone marrow cell infections, cell transplantations and hematopoietic analyses

The *Stat5a*-pMSCV-IRES-GFP retroviral vector was used to obtain viral particles and to infect cells. All mutants were obtained through PCR-mediated mutagenesis and DNA sequences were verified. Ecotopic, replication-incompetent, retroviral gpE^+^86 producer cell lines were generated and selected for high viral titers (~10^6^ particles/ml) by fluorescence-activated cell sorting (FACS). Producer cells were maintained in Dulbecco modified Eagle medium (Life Technologies, Carlsbad, CA, USA). Bone marrow (BM) was isolated from the hind limb of 12-week-old male mice (B6129F1) and cultivated (see Supplementary Methods). BM cells were co-cultured with virus producing cells and tested for infection efficiency in presence of polybrene (6 μg/ml) as described.^27^ Two million BM cells were injected into lethally irradiated female mice by tail vein injection and monitored for disease onset by white blood cell count and flow cytometry, starting at four weeks post transplant. For FACS analyses, red blood cell lysis was performed in lysis buffer (150 mM NH_4_Cl, 1 mM KHCO_3_, 0.1 mM Na_2_EDTA; pH=7.3). Single cell suspensions were prepared from BM and spleen. Cells were stained with antibodies conjugated to phycoerythrin or APC (BD Bioscience, Franklin Lakes, NJ, USA) and analyzed with Becton-Dickinson FACScalibur. Immunohistochemistry of liver, spleen and lymph node sections was performed and cells were analyzed by histo-pathology as described.^27^ Blood smears were stained with Benzidine.^28^ The mouse studies were performed in accordance to ethical approval by Austrian government authorities according to license BMWF-66.009/0282-II/3b/2012.

### T-cell rescue assay

Splenic T-cells from 12-week-old wild type (wt) and STAT5^ΔN/ΔN^ mice without splenomegaly were isolated and transferred into culture as described.^29^ Isolated splenic T-cells were co-cultured on viral producer cells as described^27^ and stimulated with anti-CD3 antibody (145.2C11) (BD Pharmingen, Franklin Lakes, NJ, USA). Different concentrations of interleukin (IL)-2 or IL-4 (R&D Systems, Minneapolis, MN, USA) were used for proliferation assays measured by ^3^H-thymidine (GE Healthcare, Chalfont St Giles, Buckinghamshire, UK) incorporation using a Wallac 1450 MicroBeta liquid scintillation counter (PerkinElmer Life and Analytical Science, Waltham, MA, USA) 18 h after adding 1 μCi/well.

### Wheat germ agglutinin (WGA) assay

The glycoprotein isolation kit (Thermo Scientific, Rockford, USA) was used and samples were prepared according to the manufacturer’s instruction. Pull down and flow through were used for immunoblotting.

### Statistics

Statistical calculations were performed using the GraphPad Prism 5.01 software (GraphPad Software Inc., La Jolla, CA, USA). Results are presented as means±standard deviation (s.d.). As statistical test either one-way analysis of variance or two-way analysis of variance was performed, as indicated in the corresponding legend. The experimental design and mouse numbers take into account variability our laboratory has observed through years, and will be sufficient to obtain valid results, no recipient animal was removed or excluded from analysis. Block randomization method for animal groups was performed in regard to BM donation for transplant settings of male donors and wt female recipients of B6129F1 genetic origin. Histo-pathology analysis was blinded by two board-certified pathologist evaluators.

### Additional materials and methods

Cell culture techniques, DNA-binding assay, real-time PCR, primer sequences, immunoblotting and immunoprecipitation analysis are described in Supplementary Methods.

## Results

### STAT5 is O-GlcNAcylated on threonine 92 in many human cancer cells

**Figure 1:**
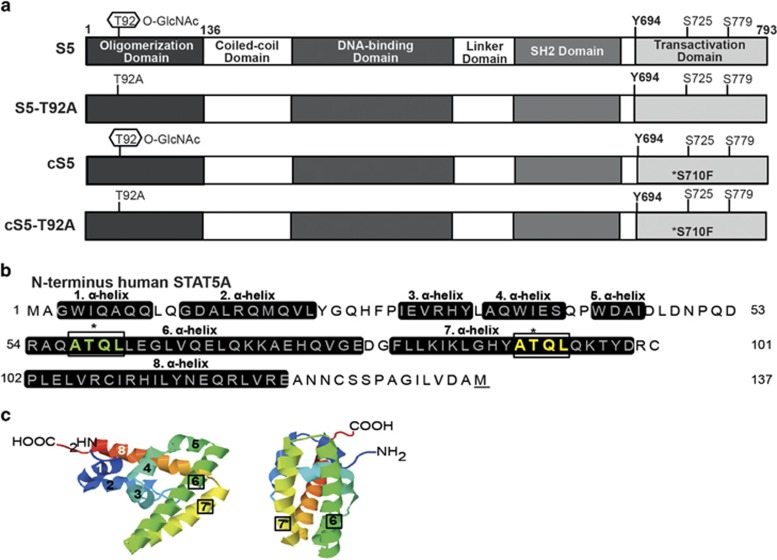
O-GlcNAcylation in the STAT5 N-terminus at position threonine 92. (**a**) Schematic representation of murine STAT5A and the mutant STAT5 proteins. (**b**) Sequence of the human STAT5A N-terminus from amino-acid 1 to 136 with the prediction for the position of the eight alpha helices. (**c**) The crystal structure of the STAT4 N-terminus in two different orientations. The numbers indicate the eight helices as shown in **b**.

STAT5A (S5) contains a threonine at position 92 (Figure 1a), which is the site of O-GlcNAcylation.^30^ This threonine is located within the N-terminal oligomerization domain of STAT5. The S5-T92A construct was mutated at position 92 and the threonine residue was replaced by alanine, preventing O-GlcNAcylation. cS5 is a hyperactive STAT5A variant, with a GOF mutation at serine 710 to phenylalanine (S710F)^8^ and was combined with the T92A variant, producing the cS5-T92A construct.

**Figure 2:**
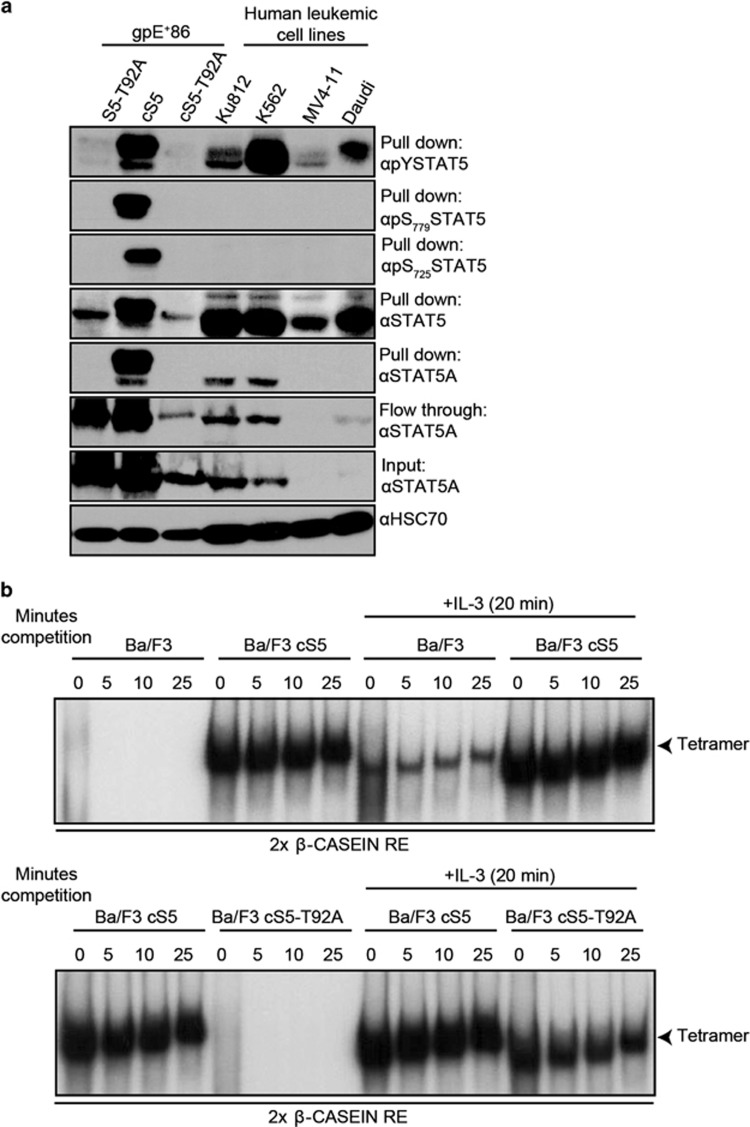
O-GlcNAcylation of STAT5 and tetramer formation. (**a**) Immunoblot of pull down and flow through from WGA assay for mouse fibroblasts gpE^+^86 and human cell lines (*n*=2). (**b**) STAT5-tetramer electrophoretic mobility shift assays (EMSAs) on a 2 × β-casein site with whole-cell extracts of parental and cS5 or cS5-T92A transfected Ba/F3 cells with and without 10 ng/ml IL-3 stimulation (*n*=2).

The sequence of the human STAT5A N-terminus from amino acid 1 to 136 (Figure 1b) was used to model the secondary structure of the STAT5 N-terminus based on the structure of the STAT4 N-terminal domain, which consists of eight alpha helices; threonine 92 is located in helix seven. An additional threonine was found in helix six at position 58 within a repeated sequence of ATQL. Interestingly, both ATQL motifs are located in close proximity to each other, representing a possible repeat motif (indicated by the boxes; Figure 1c). The threonine 58 in the first motif is conserved within the STAT family, whereas the threonine at position 92 is only conserved among the STAT5 proteins in different species (Supplementary Figure 1). To confirm the O-GlcNAcylation of threonine 92 in STAT5, mouse fibroblasts expressing different STAT5 variants were subjected to a WGA assay (Figure 2a). The lectin column, used in the WGA assay, only binds O-GlcNAcylated proteins. In addition, glycosylated proteins present in human CML (Ku812, K562), AML (MV4-11) and Burkitt lymphoma (Daudi) cell lines were tested. O-GlcNAcylated STAT5A could only be detected in the proteins pulled down from cS5-expressing fibroblasts, but not in cells expressing the T92A variant (Figure 2a). O-GlcNAcylated STAT5 is much less abundant in extracts obtained from T92A transfected cells; the residual signal is due to the expression of endogenous wt STAT5 expressed in the fibroblasts. Extracts from cS5 transfected cells exhibited much higher signal intensity. The lack of O-GlcNAcylated STAT5 in the T92A compared with the cS5 and wt STAT5 (S5) transfected gpE^+^86 cells could be shown in separate WGA assay (Supplementary Figure 2A). In the fibroblasts, tyrosine 694 and serine 725/779 phosphorylation was only detectable in the O-GlcNAcylated cS5 extracts (Figure 2a). Human leukemic cell lines, especially the CML cell lines, displayed high levels of O-GlcNAcylated STAT5. A strong STAT5 signal could be detected in proteins pulled down by WGA as well as strong tyrosine phosphorylation. AML and Burkitt lymphoma cell lines expressed STAT5A to lower extent, but showed high levels of O-GlcNAc as detected by WGA. Serine phosphorylation was not detectable in the WGA pull down proteins of the cancer cell lines (Figure 2a). Similar results for O-GlcNAcylation in human leukemic cancer cell lines were obtained with separate WGA and extract preparation (Supplementary Figure 2B).

To confirm our results from WGA as a method for the detection of O-GlcNAcylation STAT5 molecules, we also employed a monoclonal αO-GlcNAc specific antibody (CTD110.6) in immunoblotting experiments to visualize their presence in human cancer cell lines. The specificity of the antibody was evaluated in a competitive immunoblot. Pre-incubation with 100 mM GlcNAc weakened the signal (Supplementary Figures 2C and 2D). In a positive control experiment, BSA-GlcNAc was detected as strong band. Ovalbumin served as negative control that yielded no signal (Supplementary Figure 2C). We detected a protein of ~92 kDa with O-GlcNAcylation in extracts from CML or AML cell lines (Supplementary Figures 2D and 2E). The megakaryoblastic leukemia cell line Mo7e displayed O-GlcNAcylation and tyrosine phosphorylation of STAT5 independent of GM-CSF stimulation. Stimulation of UT-7 with GM-CSF enhanced the phosphorylation signal, but did not affect the O-GlcNAcylation. Cell lines such as SET-2 and Jurkat with low or no O-GlcNAc signal, had lower tyrosine phosphorylation. These experiments show that O-GlcNAcylation of STAT5 can be detected in human hematopoietic cancer cell lines and the T92A mutation causes strongly decreased O-GlcNAcylation at T92, the major glycosylation site.

### The threonine 92 to alanine mutant of STAT5 can be phosphorylated on tyrosine, retains DNA-binding ability and decreases oligomerization back to wild-type level

To investigate the functional properties of the STAT5 variant with the T92A mutation, we performed DNA-binding assays. In all, 293 T cells were transfected with the S5, cS5, S5-T92A and the cS5-T92A constructs together with the erythropoietin receptor (EpoR) followed by EPO stimulation. Immunoblots of whole-cell extracts revealed that tyrosine phosphorylation can be induced by EPO stimulation in all STAT5 variants (Supplementary Figure 3A). An electrophoretic mobility shift assay was carried out to compare the DNA-binding ability of the four constructs. All four STAT5 variants were able to form complexes with the β-casein response element and could be supershifted with a STAT5 specific antibody (Supplementary Figure 3B). This was also demonstrated in parental and transfected Ba/F3 cells, where STAT5 formed dimers under IL-3 stimulation that could be supershifted with a STAT5 antibody (Supplementary Figure 3C). Only the cS5 transfected cells were bound to the β-casein response element without IL-3 stimulation (Supplementary Figure 3C). In addition, the cS5 mutant showed high oligomer (tetramer) levels independent of IL-3 stimulation in transfected Ba/F3 cells (Figure 2b). In contrast, the T92A mutation decreased the tetramer formation to wt level, which could be increased under cytokine stimulation (Figure 2b). Upon adaption of loading to STAT5 activity levels, oligomer formation was not impaired by the T92A mutation in transfected 293 T cells (Supplementary Figure 3D). We conclude that the cS5-T92A retains its ability to specifically bind to its DNA response element, although dimer and oligomer formation are decreased back to wt level.

### O-GlcNAc deficiency counteracts the enhanced tyrosine phosphorylation of hyperactive STAT5 and reduces its target gene induction

**Figure 3:**
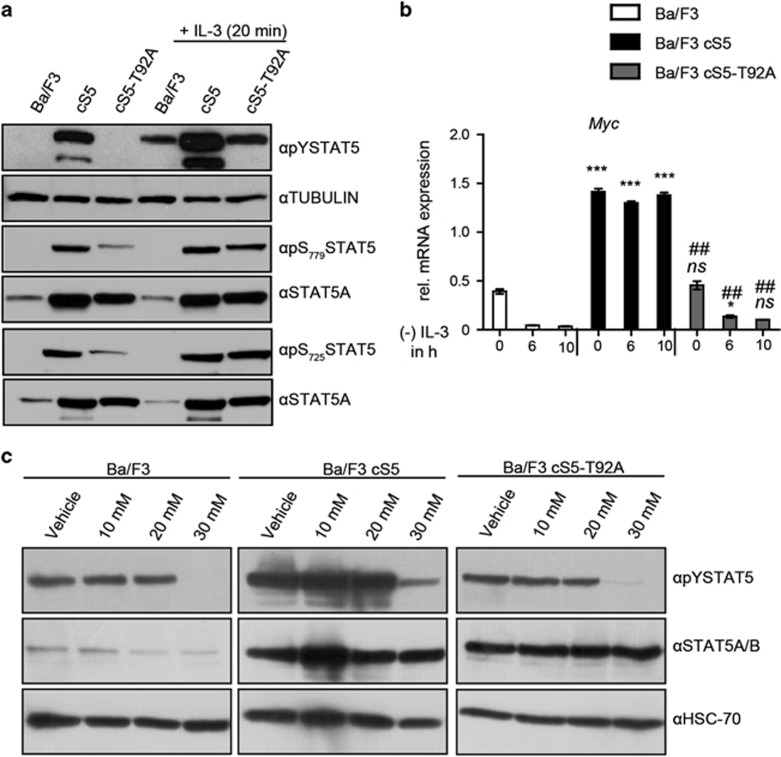
The lack of O-GlcNAcylation in STAT5A led to decreased pYSTAT5 and reduced STAT5 target gene expression in cS5 context. (**a**) pYSTAT5 and phospho-serine (pSSTAT5) immunoblots of parental and transfected (cS5 and cS5-T92A) Ba/F3 cells with and without IL-3 (20 ng/ml) stimulation (*n*=2). (**b**) Analysis of transcriptional expression of STAT5 target genes in Ba/F3 cells after IL-3 removal by semi quantitative real-time PCR with Rpl13a as housekeeping gene, performed in triplicates. Parental vs cS5 and parental vs cS5-T92A (ns=not significant; *P<0.05; **P<0.01; ***P<0.001 two-way analysis of variance (ANOVA)) cS5 vs cS5-T92A (^#^*P*<0.01; ^##^*P*<0.001 two-way ANOVA). (**c**) pYSTAT5 western blot of parental and transfected Ba/F3 cells, treated with vehicle (aqua) or incubated with 10 mM, 20 mM, 30 mM Alloxan for 1 h and stimulated with IL-3 (10 ng/ml) for 15 min (*n*=2).

We used murine Ba/F3 cells to evaluate the phospho-tyrosine levels of the STAT5 variants and measured response to IL-3 induction. As expected, Ba/F3 cells expressing the cS5 variant exhibit high pYSTAT5 levels, even in absence of IL-3 (Figure 3a). This is not the case for Ba/F3 cells expressing cS5-T92A. The T92A mutation in cS5-T92A counteracts the constitutive activation seen in cS5. cS5-T92A and the endogenous wt STAT5 present in non-transfected Ba/F3 cells can be activated by IL-3 treatment where we detected similar pYSTAT5 levels. The total STAT5 expression was comparable in cS5 and cS5-T92A transfected Ba/F3 cells. Nevertheless, higher pYSTAT5 amounts were detected in cS5 when compared with cells expressing cS5-T92A (Figure 3a). The lower tyrosine phosphorylation was also detected in a WGA pull down experiment of Ba/F3 cells cultured in presence of IL-3 (Supplementary Figure 4A). cS5-T92A-expressing cells harbor less pYSTAT5 than the un-transfected Ba/F3 cells upon IL-3 induction, when normalized to total STAT5 content. Serine 779 or 725 phosphorylation was not affected by T92A mutation (Figure 3a).

We also measured the consequences of the T92A mutation in STAT5 for its transactivation potential. STAT5 target gene mRNA expression was investigated by quantitative PCR after removal of IL-3 from growing cultures (Figure 3b and Supplementary Figure 4B). All STAT5 genes investigated were expressed significantly higher in cS5-expressing cells compared with Ba/F3 cells, even after 10 h of cytokine starvation (Supplementary Figure 4B) with the exception of *Pim1.* STAT5 target gene expression levels were similar to those found in the parental Ba/F3 cells in cS5-T92A-expressing cells and significantly decreased in cS5-T92A compared with cS5. Thus, the threonine 92 mutation neutralized the enhanced STAT5 target gene expression in cS5 upon cytokine deprivation. To further study the interaction of O-GlcNAc levels and tyrosine phosphorylation, transfected Ba/F3 cells were treated with Alloxan an OGT inhibitor.^31^ By inhibition of OGT the pYSTAT5 levels were decreased at 30 mM (Figure 3c). At the same time also the O-GlcNAcylation of the cell proteins was diminished (Supplementary Figure 5A). In addition to Alloxan, treatments with 6-diazo-5-oxo-L-norleucine were performed. The 6-diazo-5-oxo-L-norleucine inhibitor does not directly inhibit the enzyme OGT but it inhibits the glutamine fructose-6-phosphate amidotransferase in the hexosamine biosynthesis pathway. This leads to a decrease of the donor substrate synthesis of UDP-N-actylglucosamine and, therefore, to a decrease of the O-GlcNAc levels. It is commonly used to decrease the overall levels of O-GlcNAc.

When the O-GlcNAc levels were decreased under 6-diazo-5-oxo-L-norleucine treatment, also the pYSTAT5 signal was diminished in cS5-expressing cells (Supplementary Figure 5B). In contrast to STAT5A, the tyrosine phosphorylation was not diminished in 293 T cells transfected with human STAT5B with the T92A mutation compared with wt human STAT5B (Supplementary Figure 6A). Similarly, 293T transfected with a N642H GOF and T92A mutation, pYSTAT5 levels were not decreased compared with the hyperactive STAT5B N642H.

### Sustained tyrosine phosphorylation of STAT5 causes increased phospho-AKT and phospho-ERK levels

**Figure 4:**
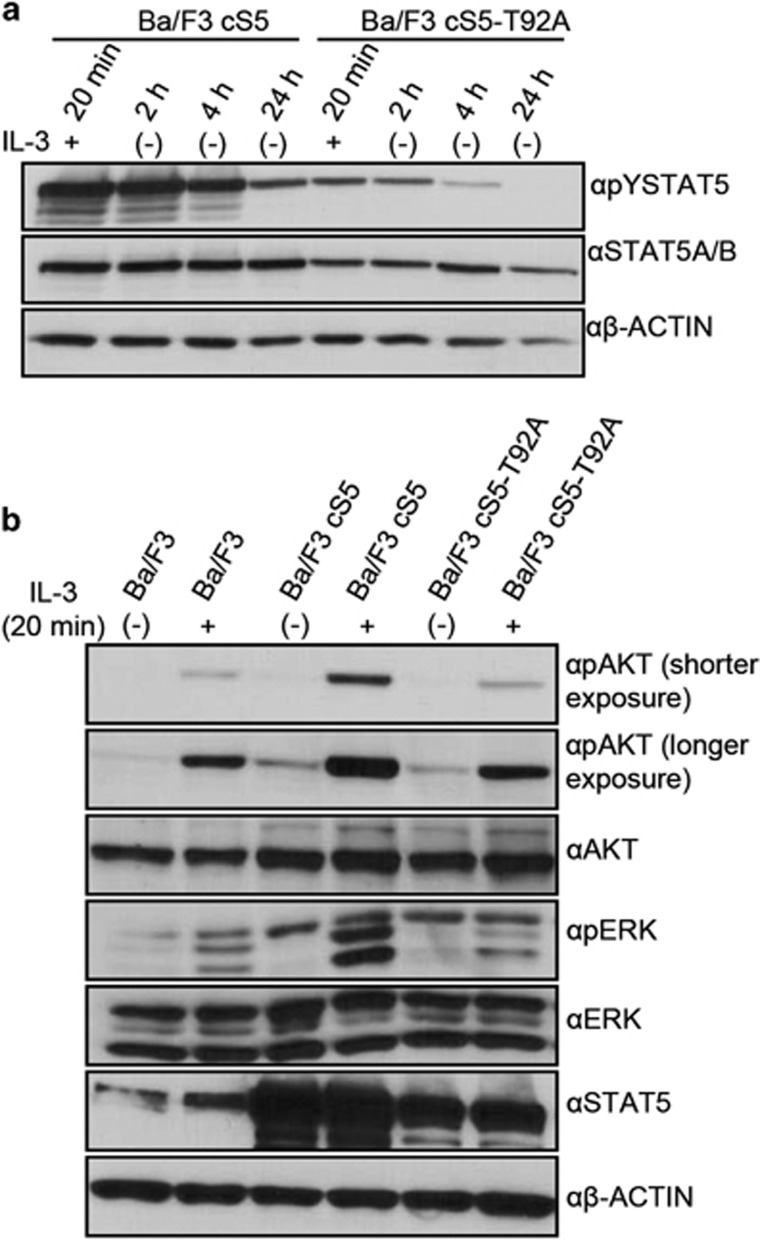
Hyperactive, O-GlcNAcylated STAT5 maintains tyrosine phosphorylation and phospho-AKT and phospho-ERK levels. (**a**) Phospho-tyrosine immunoblot of transfected Ba/F3 cells after IL-3 removal for indicated time points (*n*=2). (**b**) Phospho- and total-protein immunoblot for AKT, ERK and STAT5 in Ba/F3 cells, electroporated with cS5 or cS5-T92A (*n*=2).

Interestingly, pYSTAT5 was strongly reduced in extracts from cS5-T92A compared with cS5 transfected Ba/F3 cells (Figure 3a). To check for the time course of pYSTAT5 levels, transfected Ba/F3 cells were starved for up to 24 h after IL-3 stimulation and pYSTAT5 signal was evaluated in an immunoblot at different time points after IL-3 withdrawal. In cS5-T92A Ba/F3 cells the phosphorylation signal was already strongly reduced 4 h after IL-3 stimulation and no longer detectable after 24 h. This is in contrast to pYSTAT5 in cS5-expressing cells which remained high up to 24 h after IL-3 stimulation (Figure 4a). A comparison of the parental, cS5 and the cS5-T92A-expressing Ba/F3 cells showed that the cS5-T92A-expressing cells behaved like wt Ba/F3 cells (Supplementary Figure 6B). We have shown that cS5 can form a cytoplasmic complex with the p85 subunit of the PI3K and the scaffolding adapter protein Gab2.^32^ Thus, we tested pAKT expression levels in Ba/F3 cS5-expressing cells compared with cS5-T92A-expressing cells. Furthermore, we investigated ERK activity levels to measure downstream signaling from the IL-3 receptor-JAK2 axis after IL-3 stimulation. cS5 expression caused elevated phospho-AKT and phospho-ERK signals without IL-3 stimulation, which was further enhanced upon IL-3 stimulation, whereas cS5-T92A and wt cells displayed similar, lower phosphorylation levels (Figure 4b). Total AKT and ERK levels were similar for all Ba/F3 cell cultures. Supplementary Figure 6C shows the phospho-AKT and phospho-ERK levels relative to the total-AKT and total-ERK levels. We conclude that the O-GlcNAcylation of the hyperactive cS5 variant is required for the enhanced AKT and ERK activity.

### The loss of STAT5 O-GlcNAcylation rescues cytokine-induced growth of T-cells

**Figure 5:**
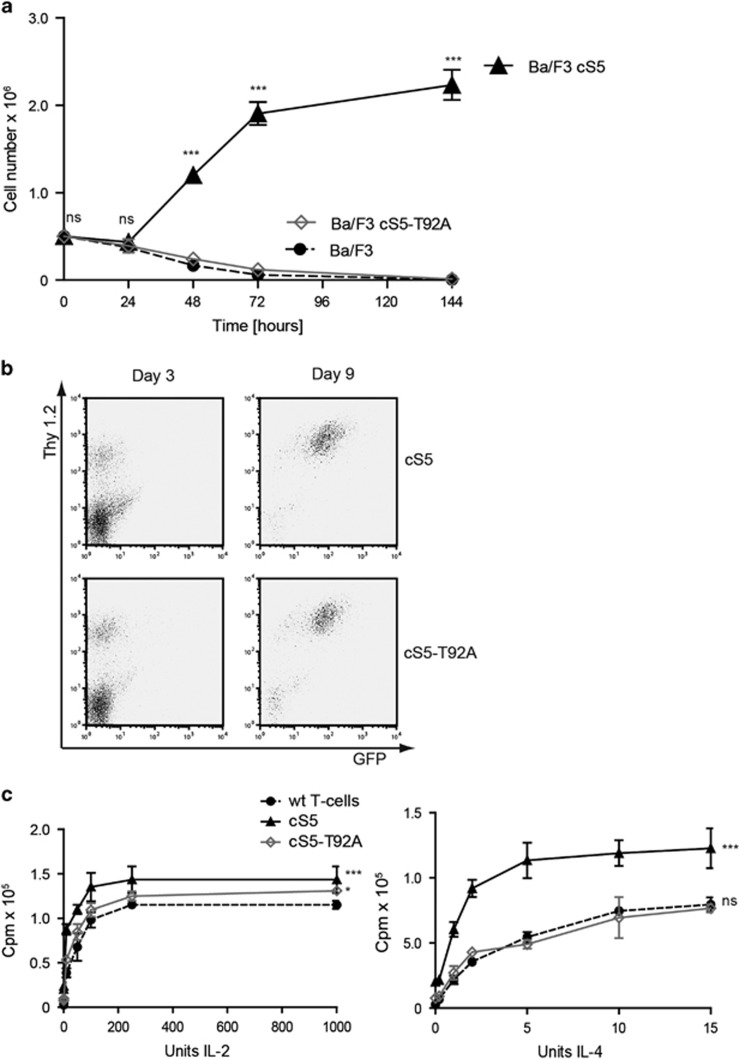
Lack of STAT5 O-GlcNAcylation in context of cS5 re-establishes cytokine dependence for its proliferative activity. (**a**) Growth curve of transfected Ba/F3 cells upon IL-3 starvation. Cells were grown and counted in triplicates (ns=not significant; ***P<0.001 two-way analysis of variance (ANOVA)) (*n*=2). (**b**) FACS analysis of transduced T-cells for Thy1.2/GFP double-positive cells (*n*=2). (**c**) Viable cells were examined for proliferation upon IL-2 and IL-4 stimulation by ^3^H-thymidine incorporation assay. Mean and s.d. were calculated from four single wells (ns=not significant; *P<0.05; ***P<0.001 one-way ANOVA) (*n*=2).

To evaluate consequences of cS5 and cS5-T92A expression on cellular phenotypes, we exploited the growth requirements of Ba/F3 cells. Wt Ba/F3 cells only survive and proliferate in presence of IL-3. In contrast, Ba/F3 cells transfected with the cS5 construct become IL-3 independent (Figure 5a). This is not the case for Ba/F3 cells expressing cS5-T92A. These cells remained IL-3 dependent (Figure 5a), confirming our observation that the lack of O-GlcNAcylation counteracts the constitutively active features of cS5.

To investigate whether lymphocytes transfected with the hyperactive STAT5 lacking the O-GlcNAcylation are still able to proliferate, we performed a T-cell rescue assay. T cells which lack the N-terminus of STAT5 did not significantly proliferate in response to stimulation with IL-2 and CD3.^29^ This defect can be rescued by introduction of a wt *Stat5* gene (Supplementary Figure 7A). cS5 or cS5-T92A transduced cells were double positive for GFP and T-cell marker Thy1.2 (Figure 5b). Both, cS5 and cS5-T92A supported proliferation upon IL-2 and IL-4 stimulation (Figure 5c), but cS5 was more potent in the enhancement of IL-4 induced proliferation. Thus, for T-cell proliferation in response to cytokines STAT5 O-GlcNAcylation is dispensable.

### The transformation potential of constitutively active STAT5 requires O-GlcNAcylation

**Figure 6:**
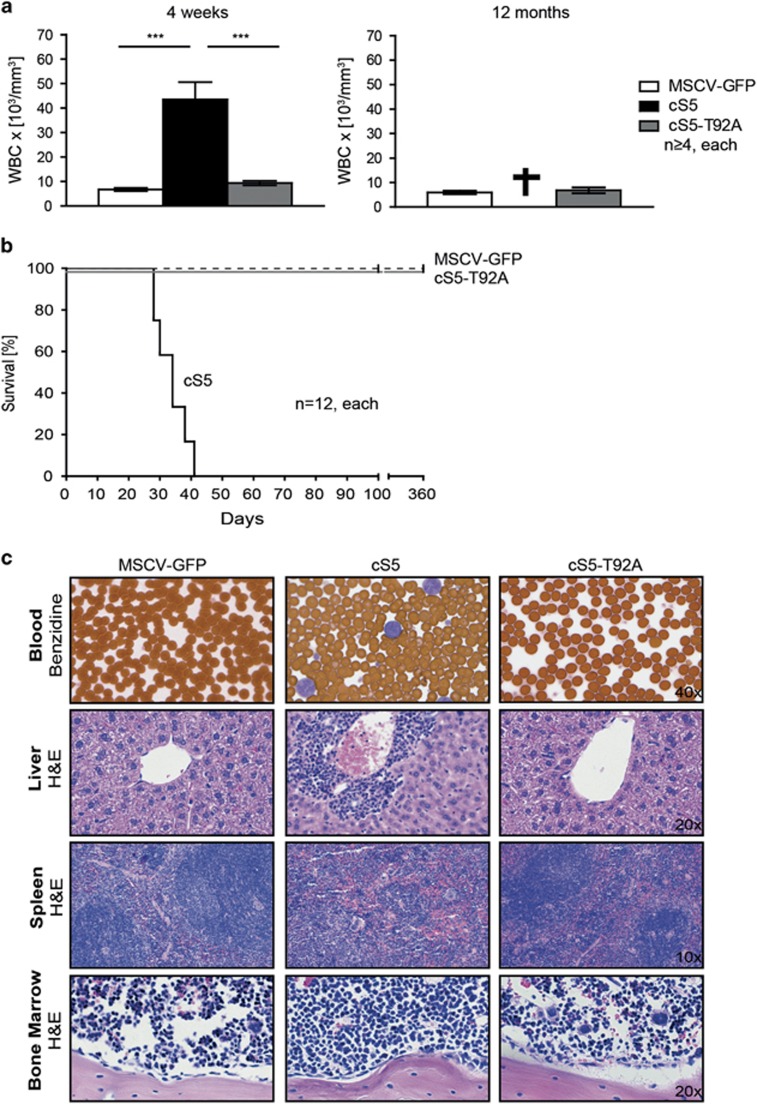
The transforming potential of cS5 requires O-GlcNAcylation. (**a**) white blood cell count of transplanted mice at 4 weeks (left) and 12 months (right) (*n*⩾4, each) (***P<0.001 one-way analysis of variance (ANOVA)) post transplant. (**b**) Kaplan–Meier blot of transplanted mice (*n*=12, each) (*P*<0.0001 log-rank test). (**c**) Histo-pathology analysis of blood, liver, spleen and BM of 4-weeks-old mice transplanted with MSCV-GFP, cS5 or cS5-T92A (Magnifications: 40 × blood; 20 × liver; 10 × spleen; 20 × BM). Blood smears were stained with Benzidine, all other organ slides were hematoxylin and eosin stained.

**Figure 7:**
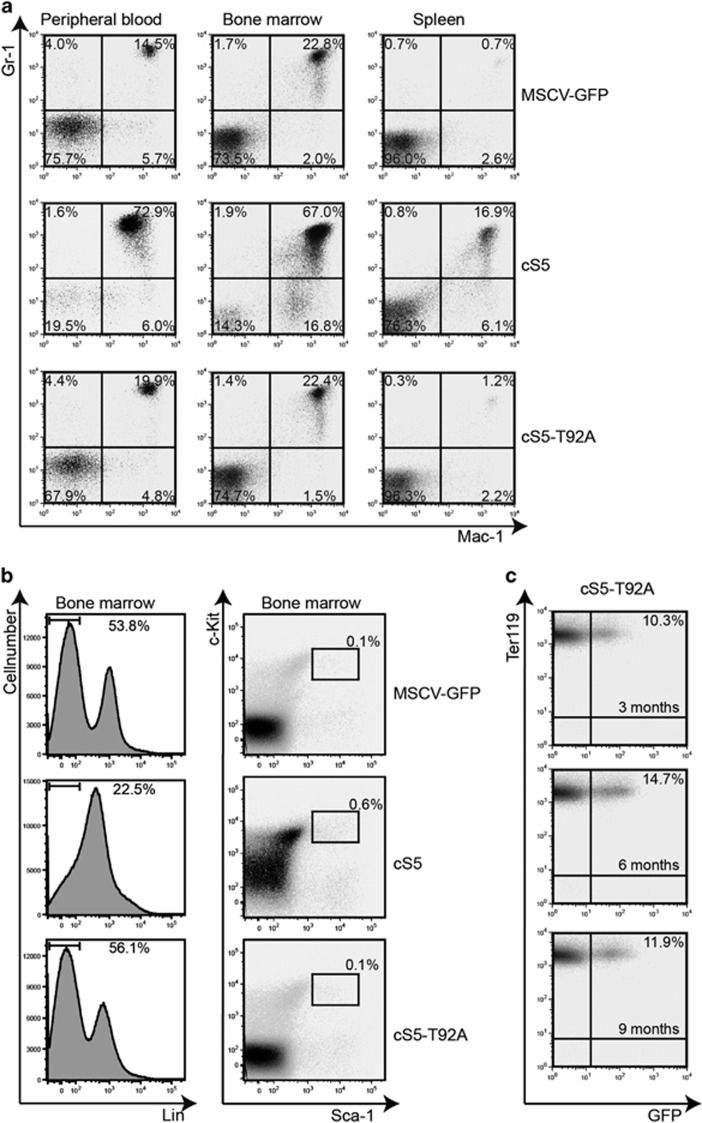
Pre-leukemic cell populations of cS5 transplanted mice showed myeloproliferative neoplasia, but cS5-T92A supports normal hematopoiesis. (**a**) Flow cytometry analysis for myeloid markers (Gr-1, Mac-1) of transplanted mice from blood, BM and spleen, 4 weeks after transplantation (*n*=4). (**b**) FACS analysis of BM from MSCV-GFP, cS5 and cS5-T92A transplanted mice for lineage-negative (Lin^-^) stem-cell antigen 1 (SCA1^+^) KIT^+^ (LSK) cells at 4 weeks post transplantation (*n*=4). (**c**) Flow cytometry analysis for Ter119 and GFP of peripheral blood from cS5-T92A transplanted mice drawn at indicated time points (*n*=4).

Next, we compared the transforming properties of cS5 to those of the cS5-T92A variant. For this purpose, the STAT5 constructs were retrovirally transduced into BM cells, transplanted into mice and development of neoplasia was monitored. Four weeks post injection, mice bearing the cS5-transduced cells showed a significantly increased white blood cell count, which was not observed in cS5-T92A transplanted mice (Figure 6a). cS5 mice had to be killed due to severe myeloproliferative disease 6 weeks post transplant, but cS5-T92A transplanted mice exhibited a white blood cell count similar to the one seen in control mice and remained healthy. The cS5-T92A mice were disease-free and alive up to 360 days (Figure 6b). In the histological analysis of blood, liver, spleen, and BM, mice transplanted with cS5 showed blasting cells in the peripheral blood, hematopoietic infiltrates in the liver, complete loss of splenic architecture, increased BM cellularity and lymphadenopathy. In contrast, cS5-T92A mice did not show any abnormalities and histologically resembled controls (Figure 6c). To further investigate the disease onset, mice transplanted with cS5-expressing cells were compared with mice transplanted with cS5-T92A-expressing cells and lineage distributions were analyzed. The cS5-transplanted mice had a high percentage of Gr-1^+^/Mac-1^+^ cells in peripheral blood, BM and spleen, whereas the cS5-T92A mice resembled vector controls (Figure 7a). In addition, FACS analysis of the BM for lineage-negative (Lin^-^) stem-cell antigen 1 (SCA1^+^) KIT^+^ (LSK) cells revealed that cS5 mice had an increased number of differentiated cells in the lineage FACS panel (Figure 7b). Interestingly, mice with cS5-T92A cell injections retained a constant GFP-positive cell fraction only in the erythroid lineage as determined at 3, 6 and 9 months post transplant, but they never developed signs of neoplasia (Figure 7c). We conclude that in context of a GOF mutation of STAT5A, loss of O-GlcNAcylation results in lost transforming ability.

## Discussion

The JAK/STAT signaling pathway connects extracellular ligands with the transcriptional machinery.^33^ Quantitative aspects of STAT signaling limit the extent and the duration of activation with decisive effects on gene expression influencing neoplasia.^13, 27^ Here, we describe a new interplay between tyrosine phosphorylation and O-GlcNAcylation of STAT5. Glycosylation at threonine 92 is a requirement for strong STAT5 tyrosine phosphorylation due to enhanced PI3K-AKT and ERK activation facilitating hematopoietic transformation.

Signaling through STAT molecules is modulated by post-translational modifications and cofactor interactions.^2^ Tyrosine phosphorylation of STAT5 activates it, but additional secondary modifications within STAT5 modulate its activity and transcriptional strength (mapped sites see www.phosphosite.org).^26^ We used the hyperactive cS5 variant to study the influence of O-GlcNAcylation on the phosphorylation state, the strength of STAT5 activation with target gene induction upon cytokine stimulation, and functional properties for transformation. Overall, STAT5 tyrosine phosphorylation and O-GlcNAcylation are functionally intertwined. Non O-GlcNAcylated STAT5 molecules can still be activated by tyrosine phosphorylation, maintain their DNA-binding activity and form tetramers, but they exhibit a much lower transactivation potential since they are not as efficient tyrosine phosphorylated and, therefore, deactivated faster by tyrosine phosphatase recycling.

It could be that diminished tyrosine phosphorylation of STAT5 and the decrease of the transforming properties in the cS5-T92A variant are associated with changes in protein stability. We exclude a severe change in protein stability of the cS5-T92A variant compared with wt STAT5A due to two different cytokine signaling read out assays: First, we performed a T-cell rescue assay with STAT5^ΔN/ΔN^ T-cells, that display severely impaired proliferation in response to IL-2 or IL-4.^29^ Both, cS5 and cS5-T92A complemented IL-2- or IL-4-diminished T-cell proliferation to similar extends. Second, upon BM transduction followed by transplantation the cS5-T92A variant remained stably expressed in the erythroid lineage up to 9 months analyzed post transplant (Figure 7c).

We link pYSTAT5 levels to the state of O-GlcNAcylation. Different scenarios could account for enhanced tyrosine phosphorylation of cS5 in comparison to non O-GlcNAcylated STAT5: First, docking of STAT5 to the IL-3 receptor as a protein dependent on UDP-GlcNAc levels itself could be impaired.^34^ Second, JAK2 kinase could bind more efficiently to O-GlcNAcylated STAT5. Third, tyrosine kinase activity might be enhanced in cS5-expressing cells. JAKs are highly phosphorylated proteins, with described negative and positive phosphorylation sites for kinase activity, where pAKT and pERK activation as downstream substrates of activated JAK2 are well known.^35^ Fourth, the reported docking and activation of cS5 to PI3K-AKT could be changed.^32^ The strong and prolonged tyrosine phosphorylation of the cS5 variant is connected to an increase of IL-3 signaling, but also absence of IL-3 stimulation displayed enhanced AKT and ERK activation. Interestingly, threonine 92 of STAT5A is not only the major site of O-GlcNAcylation^30^ but it was also described to be phosphorylated.^26^ T92 is located in a repeat of a four amino acids ATQL motif, which is also present at T58, conserved in other STATs and shown to be glycosylated.^30^ If glycosylation at T92 impacts modification at T58 and *vice versa* remains unsolved.^26^ Although, T92 is conserved among the STAT5A/B proteins, there was no significant change in tyrosine phosphorylation in the T92A mutant in human STAT5B and the hyperactive N642H derivative.^36^ Therefore, impact of O-GlcNAcylation on pYSTAT5 levels could be restricted to STAT5A. STAT5 was highly O-GlcNAcylated in human leukemic cell lines, which corroborates previous studies that discovered elevated O-GlcNAc levels in cancer cells.^37^ Colorectal and breast cancer cells contained elevated O-GlcNAc and OGT levels^37^ when compared with normal tissue. The functional significance was established in OGT knockdown experiments in breast cancer cells, which resulted in diminished proliferation.^38^ When chronic lymphocytic leukemia cells were compared with normal lymphocytes, high intracellular UDP-GlcNAc and high OGT levels were found, accompanied by higher O-GlcNAcylation of c-Myc, p53 and AKT.^39^ Excessive STAT5 activation is oncogenic in several hematopoietic malignancies such as CML, MPN, ALL or AML. STAT5 is phosphorylated by tyrosine kinases such as BCR-ABL, mutated JAK, FLT3-ITD or KIT mutations.^6, 10, 40, 41^ STAT5 inhibition led to growth inhibition and apoptosis.^41^ Expression of cS5 caused enhanced levels of STAT5 tetramers in transplant settings and mice developed myeloid neoplasia.^8^ These transforming properties of cS5 are due to the enhanced and prolonged tyrosine and serine phosphorylation, which also required the N-terminal region of STAT5 together with T92 glycosylation.^9, 27, 42, 43^ Importantly, absence of O-GlcNAcylation abolished transforming properties of cS5 and increased pYSTAT5 and tetramer levels reverted back to wt levels. Serine phosphorylation of STAT5 was not affected, indicating normal shuttling. A schematic model is shown in Supplementary Figure 7B.

The interaction of O-GlcNAcylation and tyrosine phosphorylation of STAT5 was further shown in inhibitor experiments. Tyrosine phosphorylation as well as O-GlcNAc levels were decreased under the treatment with Alloxan by inhibition of OGT.^31^ pYSTAT5 levels were also decreased by reduction of the donor substrate UDP-GlcNAc under 6-diazo-5-oxo-L-norleucine treatment. Both inhibitor treatments underline the correlation of O-GlcNAcylation and pYSTAT5, although off-target effects under inhibitor treatment cannot be excluded. Thus, future studies should perform direct deletion of OGT, for example, through CRISPR-CAS9 editing.

Functional interactions between glycosylation and phosphorylation have previously been described and the same threonine residues could be modified by both post-translational processes.^44, 45^ For OGT itself it is known, that besides nutritional sensing via UDP-GlcNAc levels, also tyrosine phosphorylation and O-GlcNAcylation control its activity.^46^ c-Myc is O-GlcNAcylated at T58, a mutational hotspot in lymphomas and this threonine can also be phosphorylated.^47^ In prostate cancer, c-Myc is stabilized by O-GlcNAcylation.^48^ OGT, O-GlcNAcase, phosphatase and kinase were found in an enzymatic complex and several kinases are described to be O-GlcNAcylated.^45^ In the colorectal cancer cell line HT29, O-GlcNAc levels were decreased by siOGT knockdown resulting in lower serine phosphorylation of PKM2.^49^ O-GlcNAcylation was recognized as a nutrient sensor, which is added to many cellular signaling proteins reversibly.^18^ Inappropriate regulation of the O-GlcNAc cycling process contributes to disease development in, for example, diabetes or cancer.^17^ This could establish a connection between energy sensing and extent of pYSTAT5 signaling. Future studies should address if O-GlcNAcylation of STAT5 could serve as a nutrient sensor controlling metabolic pathways essential for cellular physiology. Hematopoietic phenotypes, known to be regulated by STAT5 have high energy requirements. Therefore, manipulation of OGT or OGA activity might be a strategy for cancer treatment by manipulating metabolism.
