# Supplementary material for: O-GlcNAcylation of STAT5 controls tyrosine phosphorylation and oncogenic transcription in STAT5-dependent malignancies
Source: Leukemia. 2017 Feb 10;31(10):2132–42. doi: 10.1038/leu.2017.4 (PMC5629373; doi:10.1038/leu.2017.4)
Supplement: Supplementary Information [file leu20174x1.docx]

**Supplementary Methods**

**Cultivation of isolated bone marrow (BM)**

DMEM supplemented with 10% (FBS), 10 U/mL Penicillin/, 10 µg/mL Streptomycin GIBCO [Life Technologies, Carlsbad, CA, USA], 2 mM L-glutamine, 1x MEM non-essential AAs, 1 mM sodium pyruvate, 10 mM HEPES (pH=7.3) all obtained from PAA [GE Healthcare, Chalfont St Giles, Buckinghamshire, UK] and 20 ng/ml mouse interleukin-3 (mIL-3), 50 ng/ml murine interleukin-6 (mIL-6) and 200 ng/ml murine stem cell factor (mSCF) [R&D System, Minneapolis, MN, USA] was used as indicated.

**Cell culture, stimulation and transfection**

All cell lines were cultivated under standard conditions (95% humidity, 5% CO_2_, 37°C). Human K562, Ku812, Jurkat, Daudi, MV4-11 and 293T cells were purchased from Leibniz Institute DSMZ (Braunschweig, Germany), HEL were kindly provided by Prof. Dr. Oliver H. Krämer (Mainz, Germany). SET-2, Mo7e, UT-7 were a kind gift of Prof. Dr. Peter Valent (Vienna, Austria) and the murine Ba/F3 cell lines were kindly provided by Prof. Dr. Alan D'Andrea (Boston, MA, USA). All cells were kept in RPMI1640 GIBCO except human 293T, which were cultured in DMEM and human UT-7, cultured in MEM GIBCO. All media were obtained from Life Technologies [Carlsbad, CA, USA] and supplied with 10% FBS, 10 U/ml Penicillin, 10 µg/ml Streptomycin, 2 mM L-glutamine and 2 µg/ml Ciprofloxacin [SIGMA, St. Louis, MO, USA]. Mycoplasma contamination was tested using the Venor GeM Classic Mycoplasma Detection Kit (Minerva Biolabs, Berlin, Germany). 100 ng/ml Granulocyte macrophage colony-stimulating factor (GM-CSF) [PreproTech, Rocky Hill, NJ, USA] for Mo7e and 2 ng/ml for UT-7 were added for culture. Stimulation in Mo7e and UT-7 were performed after cytokine starvation with 100 ng/ml (Mo7e) and 10 ng/ml (UT-7) for 20 min. 293T cells were co-transfected by calcium phosphate precipitation with the pMSCV-IRES-eGFP and the murine pM-EpoR expression vector and stimulated with 50 U/ml erythropoietin (EPO) [Janssen-Cilag, Beerse, Belgium] where indicated. The Ba/F3 cells were transfected by electroporation and grown in the presence of 1 or 5 ng/ml recombinant mIL-3 [ImmunoTools, Friesoythe, Germany]. For stimulation, either 10 or 20 ng/ml mIL-3 was added. For the establishment of growth curves and the time course of the phospho-tyrosine Western blots, Ba/F3 cells were washed in PBS 2x before being seeded in 6-well plates in triplicates and counted at indicated time points. Alloxan Monohydrate [SIGMA, Saint Louis, MO, USA] treatment of Ba/F3 cells was performed in 6-well plates with different concentrations for 1 h followed by IL-3 stimulation. For incubation with 6-Diazo-5-oxo-L-norleucine (DON) [SIGMA, Saint Louis, MO, USA], Ba/F3 cells were transferred into 10 cm^2^ dishes, and incubated with 50 µM or in vehicle control (aqua) without IL-3 for 18 h.

**DNA binding assay**

Native whole cell extracts from co-transfected 293T cells were tested in an electrophoretic mobility shift assay (EMSA) as described previously[^1-2^](#_ENREF_1) using the ß-casein gene promoter response element. For STAT5 supershifts, the C-17 anti STAT5 C-terminus (sc-835) [Santa Cruz, Dallas, TX, USA] antibody was applied. The ß-casein response element was used alone or in tandem assaying STAT5 dimer or tetramer formation.

**RNA isolation and real-time PCR**

For the semi quantitative real-time PCR, total RNA was isolated by TriZol [Life Technologies, Carlsbad, CA, USA] extraction. For cDNA synthesis the RevertAid™ H Minus First Strand cDNA Synthesis Kit [Thermo Scientific, Rockford, USA] was used according to the manufacturer`s instruction. For the primer sequences see Supplementary Table 1. Real-time PCR was performed in triplicates on an Eppendorf Master-cycler RealPlex (Eppendorf, Hamburg, Germany). Results were quantified using the Delta C(T) method.[^3^](#_ENREF_3)

**Supplementary Table 1**

The following primers were used:

| Gene | Forward primer | Reverse primer |
| --- | --- | --- |
| Bcl2 | 5´-ACTGAGTACCTGAACCGGCATC-´3 | 5’-GGAGAAATCAAACAGAGGTCGC-3 |
| Bcl2l1 | 5’-TTGGATGGCCACCTATCTGAAT-3’ | 5’-TCTCGGCTGCTGCATTGTT-3’ |
| Ccnd2 | 5´-AGAAGGGGCTAGCAGATGA-3´ | 5´-AGGATGATGAAGTGAACACA-3´ |
| Osm | 5’-ATCGTGGCTGCTCCAACTCTT-3’ | 5’-TCAGGTTTTGGAGGCGGAT-3´ |
| Pim1 | 5’-TTCTCCACCGCGACATCAA3’ | 5’-TAGCGAATCCACTCTGGAGGAC-3’ |
| Myc | 5’-GTGCTGCATGAGGAGACACCG-3’ | 5’-ATGGAGATGAGCCCGACTCCG-3’ |
| Rpl13a | 5´-TTCTCCTCCAGAGTGGCTGT-3´ | 5´-GGCTGAAGCCTACCAGAAAG-3´ |

# Immunoblotting and immunoprecipitation

# Sample preparation and Western blotting was performed using standard techniques. The nitrocellulose membrane (0.45 µm Amersham Protran 10600002) [GE Healthcare, Chalfont St Giles, Buckinghamshire, UK] was treated for some blots with Pierce™ Western Blot Signal Enhancer [Thermo Scientific, [Waltham](https://www.google.at/search?client=firefox-a&hs=Y1o&rls=org.mozilla:de:official&channel=sb&q=waltham+massachusetts&stick=H4sIAAAAAAAAAGOovnz8BQMDgwsHnxCXfq6-gVFBiYFJmhIHiJ1RZWyqpZWdbKWfX5SemJdZlViSmZ-HwrHKSE1MKSxNLCpJLSpmPjuxre9_w_6WwAi14G83_EI2xc0AAPNtxrZhAAAA&sa=X&ei=zMBsVPiWMMX3O9SSgOAH&ved=0CJUBEJsTKAIwDw), [MA](https://www.google.at/search?client=firefox-a&hs=Y1o&rls=org.mozilla:de:official&channel=sb&q=massachusetts&stick=H4sIAAAAAAAAAGOovnz8BQMDgysHnxCXfq6-gVFBiYFJmhIniG2abV6cpKWVnWyln1-UnpiXWZVYkpmfh8KxykhNTCksTSwqSS0q9qnfeleq9_H1E1f-1d6TXRQyMUfnGgCv4IEaYgAAAA&sa=X&ei=zMBsVPiWMMX3O9SSgOAH&ved=0CJYBEJsTKAMwDw), [USA](https://www.google.at/search?client=firefox-a&hs=Y1o&rls=org.mozilla:de:official&channel=sb&q=usa&stick=H4sIAAAAAAAAAGOovnz8BQMDgysHnxCXfq6-gVFBiYFJmhIniG2ZbF5uoKWVnWyln1-UnpiXWZVYkpmfh8KxykhNTCksTSwqSS0qZnx6bfobfkahssjDH3elcUtX_pj6CwAUvWAoYgAAAA&sa=X&ei=zMBsVPiWMMX3O9SSgOAH&ved=0CJcBEJsTKAQwDw)] followed by primary antibody incubation over night at 4°C. The following antibodies were used: Anti-phospho-STAT5 (Y694) (71-6900) [Invitrogen, Camarillo, CA, USA]; anti-phospho-serine as described in Friedbichler et al[^4^](#_ENREF_4), anti-STAT5 (610191) [BD Transduction Laboratories, Franklin Lakes, NJ USA], anti-phospho-p44/42 MAPK (Erk1/2) (4370), anti-phospho-AKT, anti-p44/42 MAPK (Erk1/2) (4695), anti-AKT1/2/3 (sc-8312), anti-HSC70 (sc-7298) from [SANTA CRUZ, Dallas, TX, USA], anti-ß-ACTIN (A 5316) and anti-Tubulin (T5168) from [SIGMA, Saint Louis, MO, USA]. The membranes were incubated with HRP-conjugated secondary antibody [GE Healthcare, Chalfont St Giles, Buckinghamshire, UK] for 45 min at room temperature. For detection of O-GlcNAc CTD110.6 (MMS-248R) [COVANCE, Princeton NJ, USA] was used. For immunoprecipitation (IP) 50 µl of blocked protein A Sepharose CL-4B beads [GE Healthcare, Chalfont St Giles, Buckinghamshire, UK] were incubated over night at 4°C with 500 µg and 1 µl of CDT110.6.

**Supplementary Figure 1. Sequence comparison among STAT family members and position of relevant O-GlcNAcylation motifs in the N-terminus**

Sequence alignment of the N-termini of the mouse STAT5A/B (mSTAT5A/mSTAT5B) and human STAT (hSTAT) family members. The boxes and bold written AAs depict the position of the ATQL motive. N-terminal alignment with protein BLAST from the NCBI homepage was used to calculate identities.[^5^](#_ENREF_5) Crystal structure of the STAT4 N-terminus depicted as helical structure model. Locations of the AAs at position 92, 58 and 54 in STAT4 N-terminus are indicated.

**Supplementary Figure 2. Detection of O-GlcNAcylation in STAT5**

**(A)** Western blot of WGA assay of pull down and input from murine fibroblast cell line gpE^+^86 transfected with empty vector (GFP), wt STAT5 (S5) and STAT5 constructs (cS5; cS5-T92A) (n=1). **(B)** Western blot of WGA flow through and pull down from Ba/F3 cells transfected with cS5 and human T-ALL (Jurkat), CML (Ku812), AML (MV4-11) and Burkitt lymphoma (Daudi) cell lines (n=1). **(C)** Human and murine cell lines were immunoblotted with the O-GlcNAc antibody or the O-GlcNAc antibody with 100 mM O-GlcNAc [SIGMA, Saint Louis, MO, USA]. Ovalbumin [InvivoGen, San Diego, CA, USA] served as negative control and N-Acetylglucosamine derivatized bovine serum albumin [Vector Laboratories, Burlingame, CA, USA] as positive control (n=1). **(D)** O-GlcNAc Western blot of unstimulated human cell lines and cell lines stimulated with GM-CSF (+), with and without 100 mM GlcNAc. (n=1). **E)** Immunoblot of human leukemic cell lines for O-GlcNAc, pYSTAT5 and STAT5 protein levels (n=2).

**Supplementary Figure 3. STAT5 without O-GlcNAcylation retains residual functional activity**

**(A)** 293T cells were co-transfected with the erythropoietin receptor and the STAT5 constructs. After two days, cells were treated with 50 U/ml erythropoietin (Epo) (+) for 20 min or left untreated (-). Whole cell extracts where subjected to immunoblot analysis (n=2). **(B)** Epo stimulated (+) and unstimulated extracts (-) from **(A)** were subjected to EMSA on the ß-casein site with or without STAT5 supershift antibody (n=2). **(C)** EMSA of whole cell extracts of parental and cS5 or cS5-T92A transfected Ba/F3 cells with and without IL-3 stimulation (10 ng/ml) on a single ß-casein site with or without STAT5 supershift antibody (n=2). **D)** Epo stimulated whole cell extracts of 293T cells transfected with the STAT5 constructs were subjected to STAT5-tetramer EMSAs on a 2x ß-casein site with loading adjusted to activity levels (n=2).

**Supplementary Figure 4. O-GlcNAcylation of STAT5 and target gene expression**

**(A)** Western blot of pull down and flow through of WGA assay from parental Ba/F3 cells harvested during IL-3 culture conditions, with wt STAT5 (Ba/F3S5) and STAT5 constructs (Ba/F3S5-T92A; Ba/F3cS5; Ba/F3cS5-T92A) transfected Ba/F3 cells (n=1). **(B)** Transcriptional expression of STAT5 target genes in Ba/F3 cells after IL-3 removal by semi quantitative real-time PCR with Rpl13a as housekeeping gene, performed in triplicates. Parental vs cS5 and parental vs cS5-T92A (ns=not significant; *=p<0.05; **=p<0.01; ***=p<0.001 2-way ANOVA) cS5 vs cS5-T92A (#=p<0.01; ##=p<0.001 2-way ANOVA).

**Supplementary Figure 5. O-GlcNAc and phospho-tyrosine modifications of STAT5 in Ba/F3 cells after inhibitor treatment**

**(A)** CTD110.6 Western blot for O-GlcNAc detection of parental and transfected Ba/F3 cells, treated with vehicle (aqua) or incubated with 10 mM, 20mM, 30mM Alloxan for 1 h and stimulated with IL-3 (10 ng/ml) (+) of left unstimulated for 15 minutes (n=2). **(B)** Western blot of parental and transfected Ba/F3 cells, 18 h IL-3 starved, left untreated, treated with vehicle (aqua) or incubated with 50 µM DON (n=2).

**Supplementary Figure 6. Phospho-tyrosine of STAT5 and phospho-AKT and phospho-ERK levels in Ba/F3 cells**

**(A)** 293T cells were transfected with empty vector (pMSCV), wt human STAT5B (h5B) and with h5B harboring different mutations. Cells were starved for 3 h in 1% FBS followed by growth hormone [ImmunoTools, Friesoythe, Germany] stimulation (500 ng/ml) 20 min (+) or left unstimulated (-). Cell extracts were subjected to a pYSTAT5 immunoblot (n=2). **(B)** Time course of pYSTAT5 for parental and transfected Ba/F3 cells analyzed in an immunoblot (n=2). **(C)** Quantification of relative phospho-AKT and phospho-ERK levels compared to total AKT and total ERK levels from Figure 4B using ImageJ 1.48V and GraphPad Prism 5.01 software. (+) with 20 min of IL-3 stimulation.

**Supplementary Figure 7. Scheme of T-cell rescue assay and Summary of O-GlcNacylation of STAT5**

**(A)** Scheme of T-cell rescue assay. Splenic STAT5^ΔN/ΔN^ T-cells, which are unable to significantly proliferate in response to IL-2 and CD3 activation, compared to wt T-cells, reconstituted with cS5 and cS5-T92A STAT5 variants. **(B)** Schematic representation of the hexosamine biosynthesis pathway followed by O-GlcNacylation of cS5 (with serine 710 to phenylalanine mutation) with high pYSTAT5 and oligomerization levels and cS5-T92A without O-GclNAcylation of threonine 92 leading to low pYSTAT5 and oligomerization levels. 6-Diazo-5-oxo-L-norleucine (DON) (yellow box) interferes with the donor substrate synthesis of UDP-N-actetylglucosamine at the enzyme Glutamine fructose-6-phosphate amidotransferase (GFAT) to decrease O-GlcNAc levels and Alloxan (yellow box) inhibits OGT to reduce O-GlcNAcylation.

**References**

1. Kornfeld JW, Grebien F, Kerenyi MA, Friedbichler K, Kovacic B, Zankl B*, et al.* The different functions of Stat5 and chromatin alteration through Stat5 proteins. *Frontiers in bioscience : a journal and virtual library* 2008; **13:** 6237-6254.

2. Moriggl R, Sexl V, Kenner L, Duntsch C, Stangl K, Gingras S*, et al.* Stat5 tetramer formation is associated with leukemogenesis. *Cancer cell* 2005; **7:** 87-99.

3. Livak KJ, Schmittgen TD. Analysis of relative gene expression data using real-time quantitative PCR and the 2(-Delta Delta C(T)) Method. *Methods* 2001; **25:** 402-408.

4. Friedbichler K, Kerenyi MA, Kovacic B, Li G, Hoelbl A, Yahiaoui S*, et al.* Stat5a serine 725 and 779 phosphorylation is a prerequisite for hematopoietic transformation. *Blood* 2010; **116:** 1548-1558.

5. Altschul SF, Gish W, Miller W, Myers EW, Lipman DJ. Basic local alignment search tool. *Journal of molecular biology* 1990; **215:** 403-410.
