# Supplementary figures and images for: O-GlcNAcylation of STAT5 controls tyrosine phosphorylation and oncogenic transcription in STAT5-dependent malignancies
Source: Leukemia. 2017 Feb 10;31(10):2132–42. doi: 10.1038/leu.2017.4 (PMC5629373; doi:10.1038/leu.2017.4)

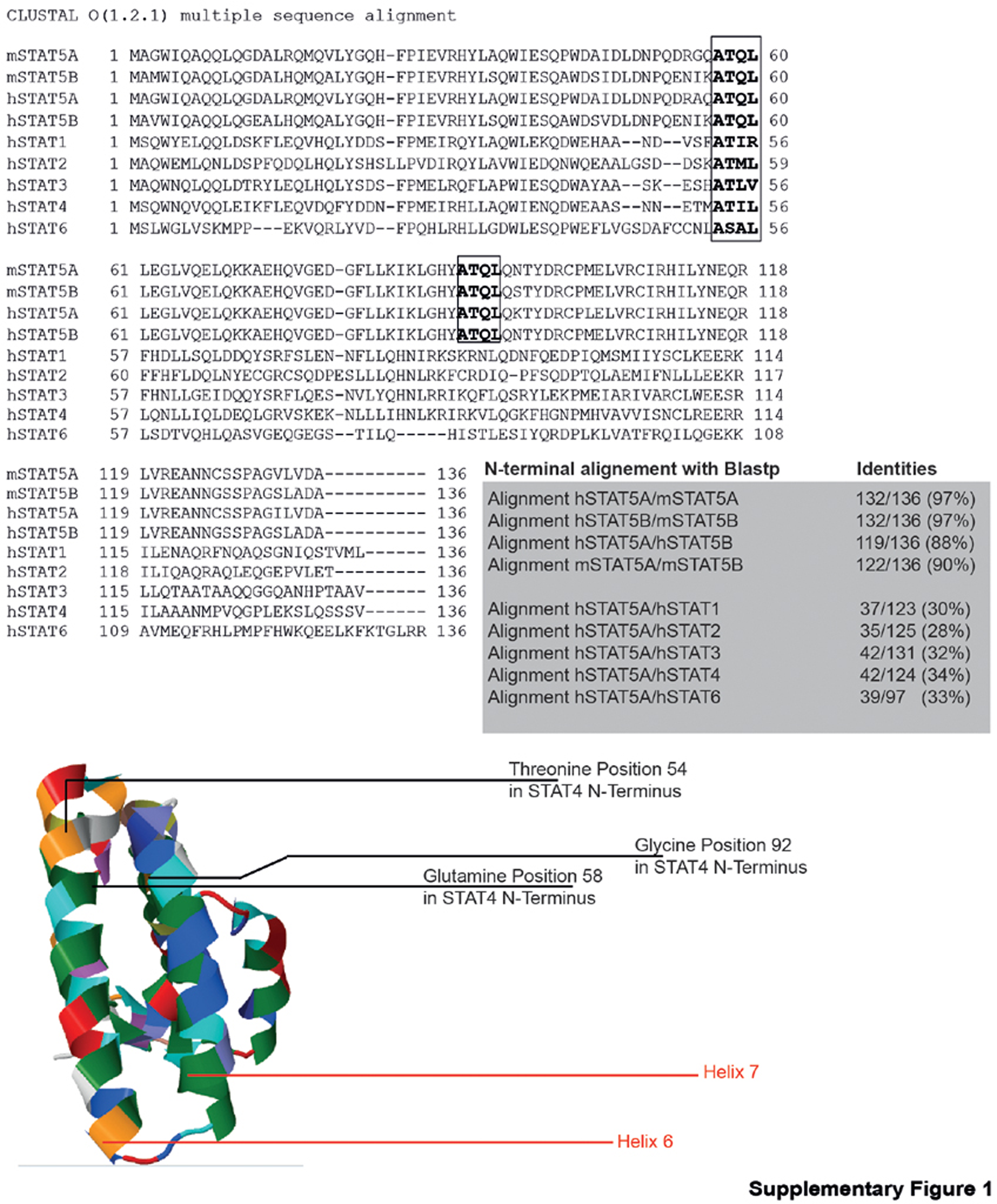

Supplement: Supplementary Figure S1 [file leu20174x3.tif]

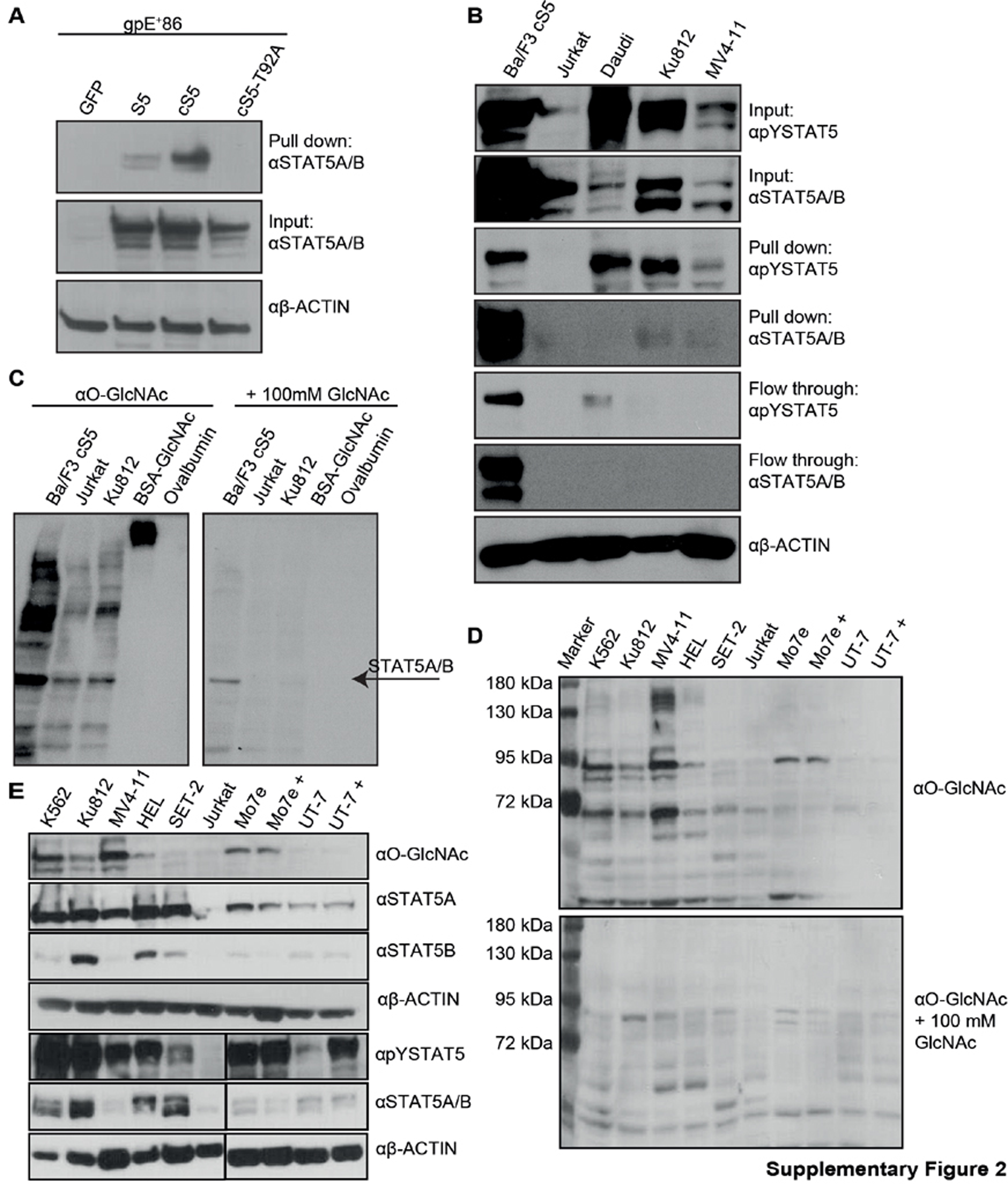

Supplement: Supplementary Figure S2 [file leu20174x4.tif]

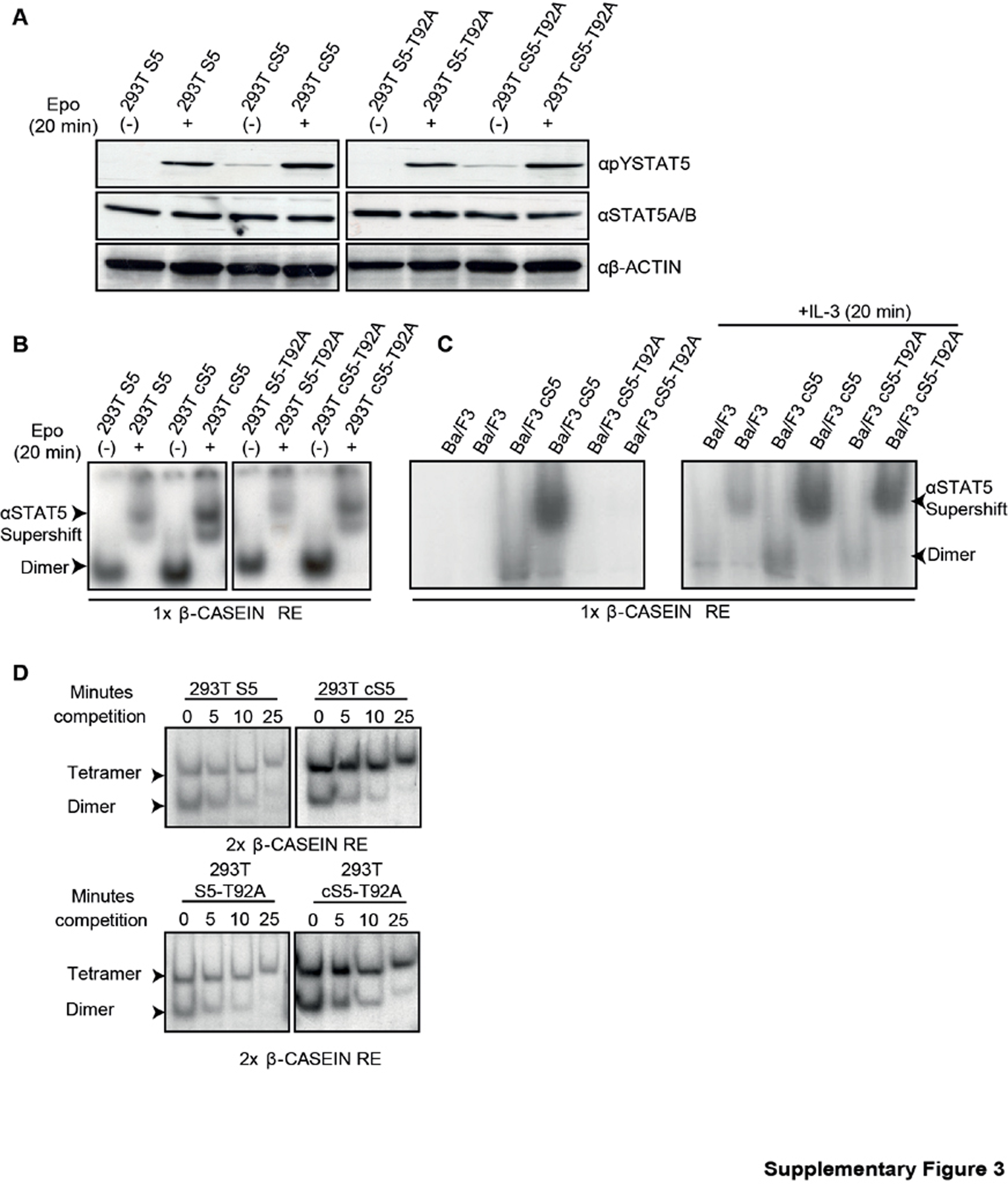

Supplement: Supplementary Figure S3 [file leu20174x5.tif]

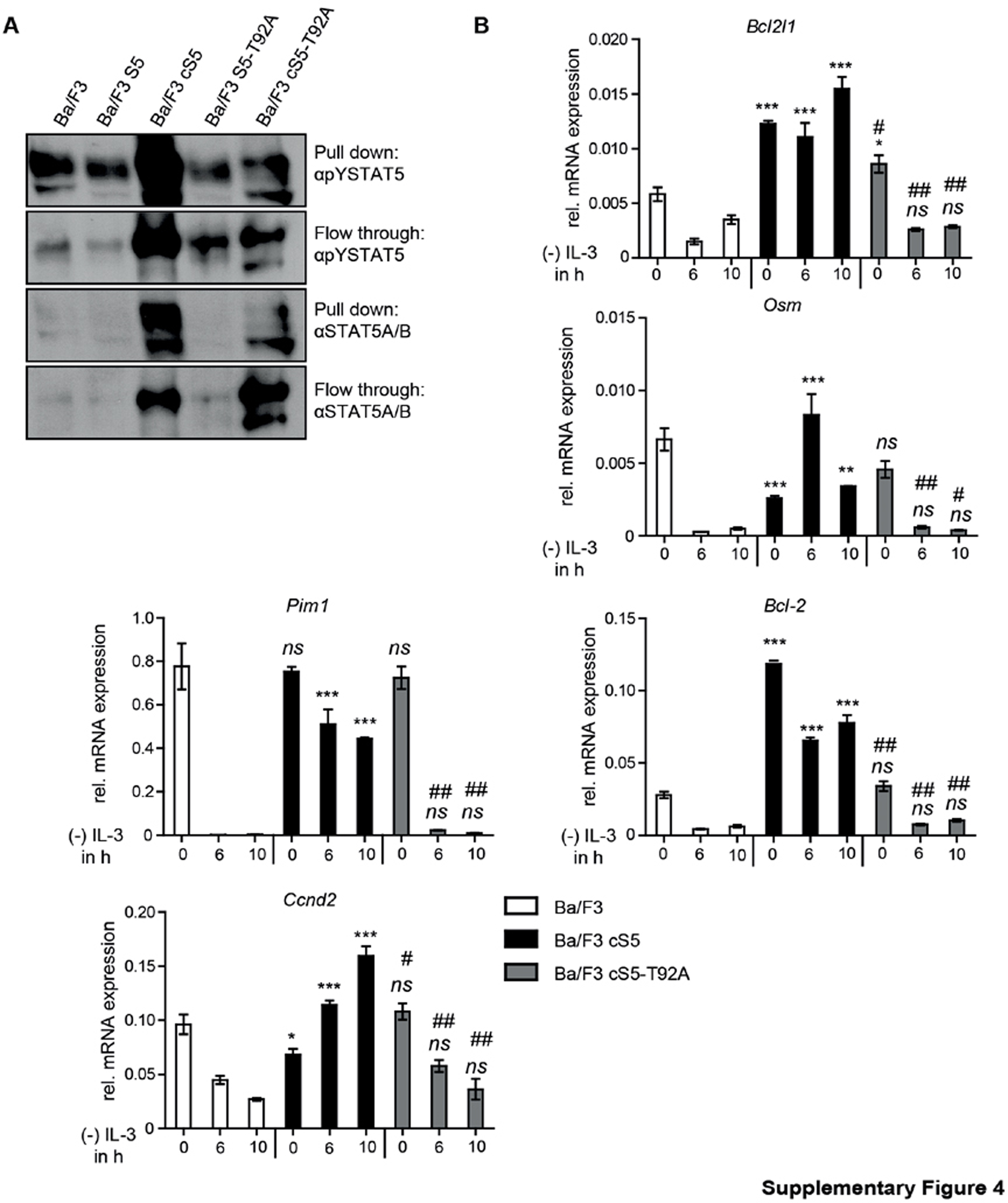

Supplement: Supplementary Figure S4 [file leu20174x6.tif]

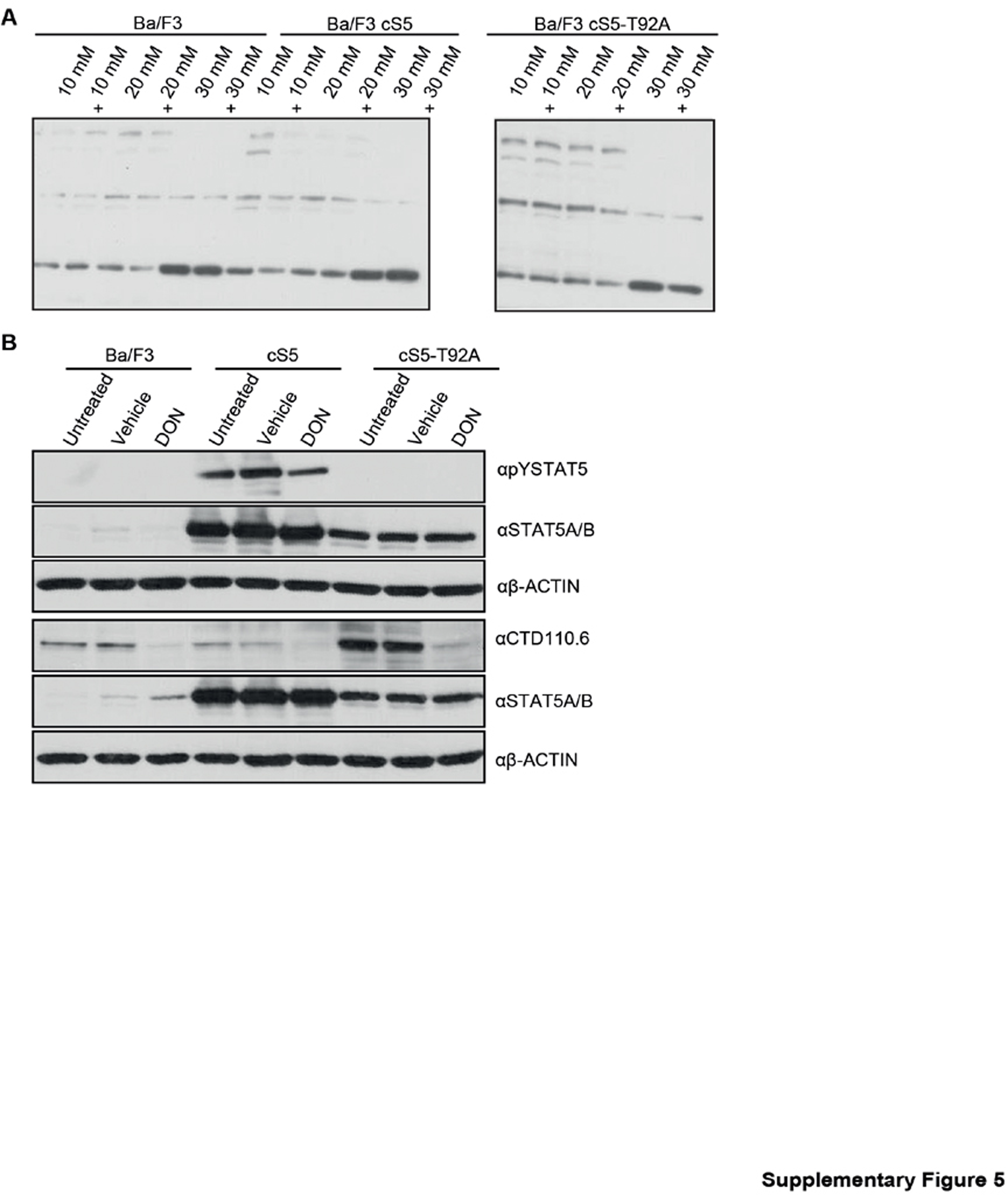

Supplement: Supplementary Figure S5 [file leu20174x7.tif]

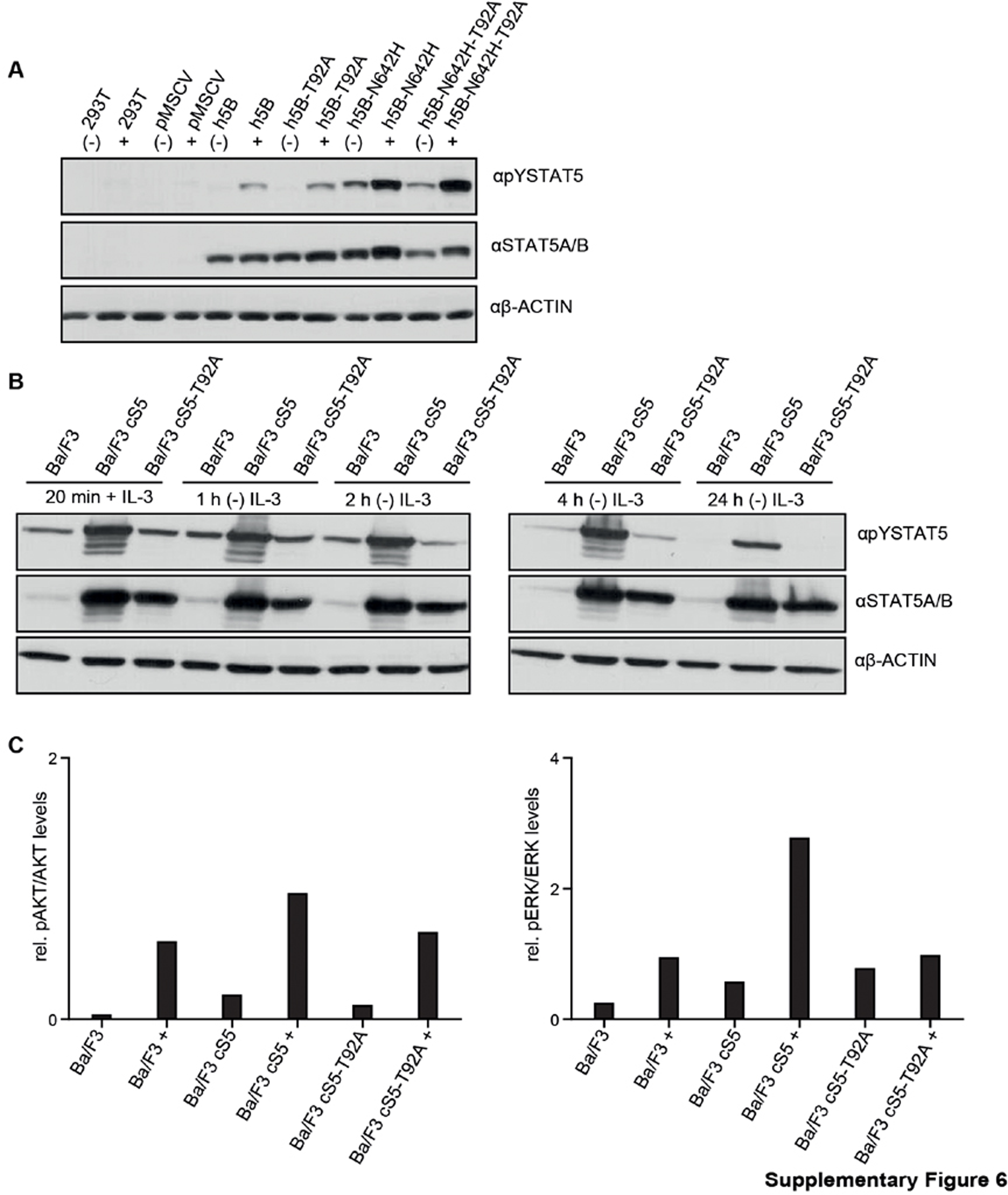

Supplement: Supplementary Figure S6 [file leu20174x8.tif]

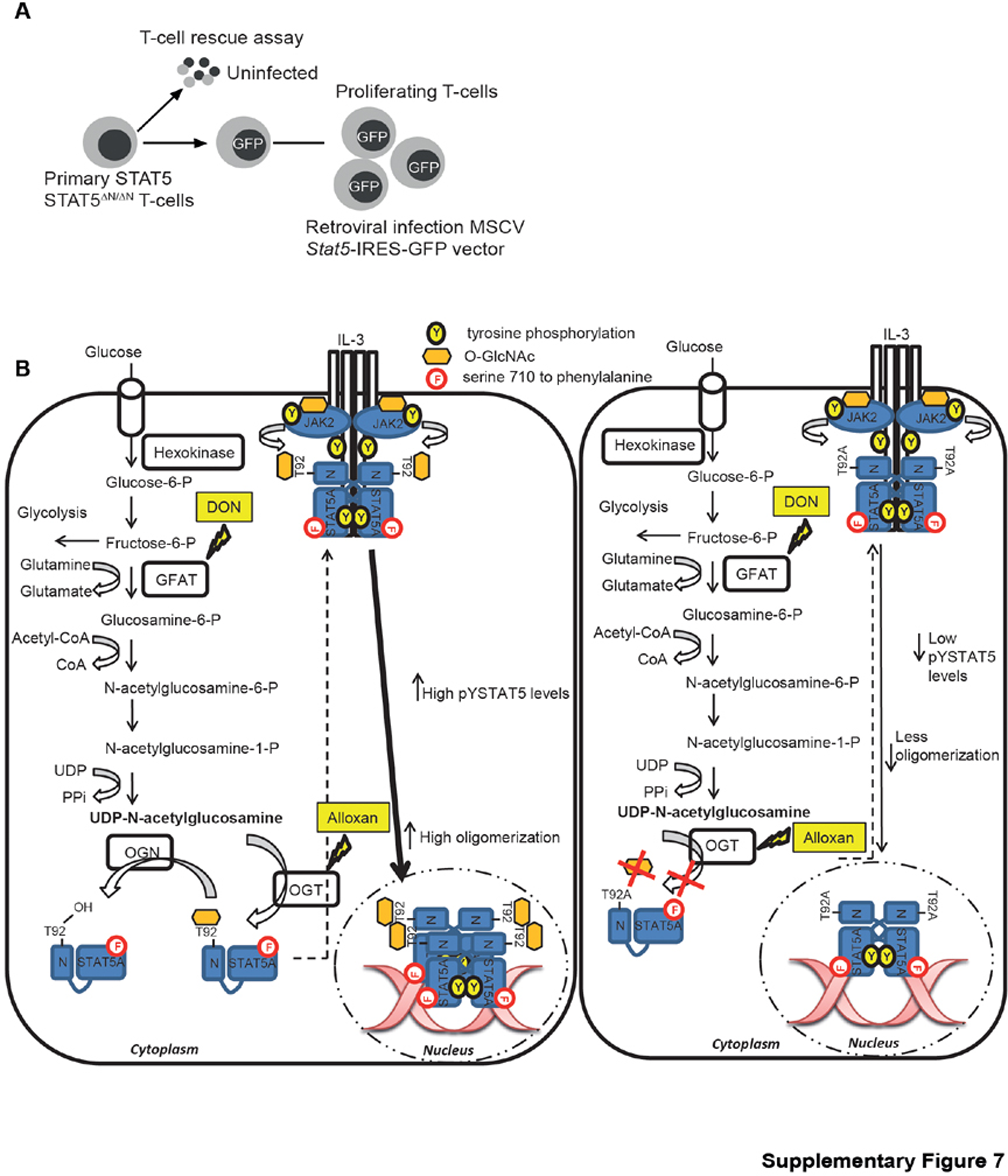

Supplement: Supplementary Figure S7 [file leu20174x9.tif]
